# Effects of Guava (*Psidium guajava* L.) Leaf Extract on the Metabolomics of Serum and Feces in Weaned Piglets Challenged by *Escherichia coli*

**DOI:** 10.3389/fvets.2021.656179

**Published:** 2021-05-24

**Authors:** Dingfa Wang, Luli Zhou, Hanlin Zhou, Guanyu Hou

**Affiliations:** Tropical Crops Genetic Resources Institute, Chinese Academy of Tropical Agricultural Sciences, Haikou, China

**Keywords:** weaned piglets, metabolomics, enterotoxigenic *Escherichia coli*, guava leaf extract, intestinal barrier function

## Abstract

The effects of dietary supplementation with guava leaf extracts (GE) on intestinal barrier function and serum and fecal metabolome in weaned piglets challenged by enterotoxigenic *Escherichia coli* (ETEC) were investigated. In total, 50 weaned piglets (Duroc × Yorkshire × Landrace) from 25 pens (two piglets per pen) were randomly divided into five groups: BC (blank control), NC (negative control), S50 (supplemented with 50 mg kg^−1^ diet GE), S100 (100 mg kg^−1^ diet GE), and S200 (200 mg kg^−1^ diet GE), respectively. On day 4, all groups (except BC) were orally challenged with enterotoxigenic ETEC at a dose of 1.0 × 10^9^ colony-forming units (CFUs). After treatment for 28 days, intestinal barrier function and parallel serum and fecal metabolomics analysis were carried out. Results suggested that dietary supplementation with GE (50–200 mg kg^−1^) increased protein expression of intestinal tight junction proteins (ZO-1, occludin, claudin-1) (*p* < 0.05) and Na^+^/H^+^ exchanger 3 (NHE3) (*p* < 0.05). Moreover, dietary supplementation with GE (50–200 mg kg^−1^) increased the level of tetrahydrofolic acid (THF) and reversed the higher level of nicotinamide-adenine dinucleotide phosphate (NADP) induced by ETEC in serum compared with the NC group (*p* < 0.05), and enhanced the antioxidant capacity of piglets. In addition, dietary addition with GE (100 mg kg^−1^) reversed the lower level of *L*-pipecolic acid induced by ETEC in feces compared with the NC group (*p* < 0.05) and decreased the oxidative stress of piglets. Collectively, dietary supplementation with GE exhibited a positive effect on improving intestinal barrier function. It can reprogram energy metabolism through similar or dissimilar metabolic pathways and finally enhance the antioxidant ability of piglets challenged by ETEC.

## Introduction

Weaned piglets infected with enterotoxigenic *Escherichia coli* (ETEC) may cause post-weaning diarrhea, which leads to growth retardation and damage to the innate and adaptive immune systems of piglets. These risk factors increase the morbidity and mortality of piglets and result in large economic losses in the swine industry worldwide (1, 2). Regarding the mechanisms of ETEC infectious diarrhea, it has been demonstrated that ETEC can produce colonization factors (CFs) and enterotoxins that adhere to the intestinal mucosa of piglets, and this action inhibits intestinal immune function, perturbs hydro-electrolytic secretions in the intestine, and results in the occurrence of diarrhea (3).

As a consequence of this, veterinary antibiotics have been commonly used to treat intestinal infections for improving animal growth and health in several decades. However, concerns about antimicrobial resistance, residue accumulation in animal products, and environmental pollution have led to a limited application of antibiotics as growth promoters (4, 5). Due to these factors, searching for alternatives to antibiotic growth promoters, such as pro- and prebiotics, organic acids, enzymes, and plant extracts, have attracted more and more attention (5–7). Among the candidate alternatives to antibiotics, plant extracts appear to be one of the most widely accepted (8, 9).

Guava (*Psidium guajava* L.) is a tropical fruit and medicinal plant, which is mainly distributed in the tropical and subtropical areas. Guava leaf extract (GE), known as an herbal medicine for the treatment of respiratory and gastrointestinal diseases (10), is reported to contain phenolics, triterpenoids, and other compounds that have antibacterial, antioxidant, and anti-inflammatory activities (11–13). Pruning usually is used to stimulate growth and influence fruiting in guava (14); thus, residual guava leaves from pruned processing are promising sources of natural feed additives, which may be utilized as a potential alternative for in-feed antibiotics.

Current studies have demonstrated that GE possesses antidiarrheal activity in various diarrhea models (15, 16). Our previous study also indicated that GE could attenuate diarrhea and improve intestinal anti-inflammatory ability in piglets challenged by ETEC (17). However, there are few reports about the relationship between the antidiarrheal effect of GE and related metabolic regulation. In the post-genomic era, metabolomics is an emerging strategy of research in the field of biological sciences, which provides a platform to study the endogenous metabolite changes in response to a biological system with genetic or environmental changes (18). In fact, metabolomics may shed light on the complex interaction mechanism between the intestinal diarrhea disease and metabolic phenotype and can regulate them to obtain therapeutic benefits (19). Therefore, here, we analyzed the intervention of GE on metabolic profiling and related endogenous differential metabolites by metabolomics in weaned piglets. Meanwhile, we also evaluated the effects of GE on tight junction-related proteins in weaned piglets, aiming to provide a potential window through which to explore the crosstalk between GE-mediated metabolic changes and its antidiarrheal processes during the progression of weaned piglets challenged by ETEC.

## Materials and Methods

### Preparation of Guava Leaf Extract

Fresh guava leaves were collected from the guava plantations in Qionghai City of Hainan Province, China, during the pruning period in July 2018, and fresh leaves were dried (60°C, 24 h) and powdered. The powder (50 kg) was exhaustively extracted with 95% ethanol three times at room temperature and then filtered. The solvent was evaporated under reduced pressure using a rotary vacuum evaporator to afford GE (3.47 kg), which was stored at 4°C for an animal experiment.

### Feeding Trial and Experimental Design

The current feeding trial is from our previous published study (17). Fifty 21 ± 3 day-old crossbred weaned piglets (Duroc × Yorkshire × Landrace, 7.35 ± 0.18 kg) were selected and transported from the piggery to the barn, where they were randomly allotted to five groups of five replicate pens per group (two piglets per pen). The five groups were as follows: (1) blank control group (BC), piglets were fed diet without supplements and ETEC challenge; (2) negative control group (NC), piglets were fed diet without supplements and challenged by ETEC; (3) S50 group (S50), piglets were fed diet supplemented with 50 mg kg^−1^ GE and challenged by ETEC; (4) S100 group (S100), piglets were fed diet supplemented with 100 mg kg^−1^ GE and challenged by ETEC; (5) S200 group (S200), piglets were fed diet supplemented with 200 mg kg^−1^ GE and challenged by ETEC. The diet was formulated to meet the nutrient recommendations of the National Research Council (2012). The ingredient and nutrient composition of basal diet were presented in Supplementary Table 1.

Feed and water were available *ad libitum* during the 28-day experimental period. All piglets were housed in a weaner facility temperature maintained at 25 ± 0.5°C, with 12 h of light and dark. On day 4, all piglets (except BC) were orally challenged with about 1.0 × 10^9^ colony-forming units (CFUs) of ETEC according to the method developed by Wu et al. (20). ETEC was obtained from the China Veterinary Culture Collection Center (Beijing, China). The occurrence of diarrhea during the whole experiment for each group was observed.

### Sample Collection

On day 29, one pig was randomly selected from each pen, and the blood samples were collected from the jugular vein, and serum was prepared by centrifugation at 700 × g for 15 min at 4°C and stored at −80°C until metabolomics analysis. After sampling, all piglets were anesthetized by an intraperitoneal injection of 50 mg kg^−1^ pentobarbital sodium and were killed by exsanguination. Fecal samples were collected directly in 10-ml sterile plastic tubes from the rectum of piglets and stored at −80°C until analysis. The small intestine was removed, and a piece (4-cm length) of the middle jejunum was collected, gently rinsed with 0.1 M phosphate-buffered saline (PBS) at pH 7.2, and then fixed in 10% formaldehyde-phosphate buffer for subsequent immunohistochemical analysis.

### Immunohistochemistry

Immunohistochemical assay was used to detect the claudin-1, occludin, zonula occludens 1 (ZO-1), and Na^+^/H^+^ exchanger 3 (NHE3) proteins expression in the jejunal mucosa with densitometric analysis as described previously (21). Polyclonal primary antibodies against claudin-1, occludin, ZO-1, and NHE3 (1:200 dilution, Proteintech, Wuhan, China) were employed. The average integrated optical density of the positive products was detected by using the Image-Pro Plus software (version 6.0 for Windows) at 200 × magnification.

### Serum and Fecal Sample Preparation and Analysis by Ultra-High-Performance Liquid Chromatography Coupled With a Hybrid Quadrupole Time-of-Flight Mass Spectrometry

Serum and fecal samples were extracted prior to analysis by ultra-high-performance liquid chromatography coupled with a hybrid quadrupole time-of-flight mass spectrometry (UHPLC-QTOF-MS) in positive ionization mode. Serum samples (100 μl) were prepared via one-step protein precipitation with 400 μl of methanol (TEDIA, Fairfield, USA). The samples were left at −80°C for 6 h after a 2-min vortex. After that, the samples were centrifuged at 20,000 × g for 10 min at 4°C. Then 400 μl of supernatant was transferred into an Eppendorf Tube, and the supernatant was concentrated in a vacuum centrifugal concentrator for 1 h using SPD121P SpeedVac Concentrator (Thermo Fisher, Germany), then reconstituted with 100 μl of acetonitrile (ACN) (MERCK, Darmstadt, Germany) for UHPLC-QTOF-MS analysis as described previously (22).

For extraction of the fecal samples, fecal samples (100 mg) were prepared via protein precipitation with 500 μl of methanol. Then the samples were vigorously vortexed for 5 min and centrifuged at 20,000 × g at 4°C for 10 min. After the supernatant was collected, the residue again was extracted according to the above extraction procedure and combined with the previous supernatant. At last, 200 μl of supernatant was transferred into the sampling vial for UHPLC-QTOF-MS analysis as described previously (23).

Briefly, the prepared sample (5 μl) was injected into an XBridge HILIC (2.1 × 100 mm, 3.5 μm) column (Waters, USA) at 30°C in an LC-30AD series UHPLC system (Nexera^TM^, Shimadzu, Japan), coupled with QTOF mass spectrometer (TripleTOF5600, Sciex, USA, Concord, ON) equipped with Turbo V ESI (electrospray ionization) sources. The mass spectrum was scanned and collected (*m/z* 70–1,000) in positive mode at a flow rate of 0.25 ml min^−1^. The chromatographic gradient condition for samples analysis was 80–20% B over 0–24 min, 20–80% B over 24–24.5 min, and the composition was held at 80% B for 8.5 min, where A = 50% ACN, and 50% water contains 0.1% formic acid (SIGMA, Deisenhofen, Germany), and B = 95% ACN, and 5% water contains 0.1% formic acid.

Drying gas temperature and the ion spray voltage were set at 550°C and 5,000 V, respectively. Atomization gas pressure, auxiliary heating gas pressure, and curtain gas pressure were set at 45, 45, and 35 psi, respectively. The instrument was mass calibrated by automatic calibration infusing the Sciex Positive Calibration Solution (Sciex, Foster City, CA, USA) after every six-sample injections. One quality control sample and one blank vial were run after each cycle of 10 samples.

Automatic peak extraction, peak matching, peak alignment, and normalization preprocessing on the acquired data were performed using MarkerView software (Sciex, USA). The retention time and *m/z* tolerances were 0.1 min and 10 ppm, respectively; the response threshold was 100 counts and the isotope peak was removed. After Pareto scaling, principal component analysis (PCA), and partial least-squares discrimination analysis (PLS-DA) models were carried out to visualize the metabolic difference among BC, NC, S50, S100, and S200 groups. The quality of the models was described using R^2^X and R^2^Y. To avoid model overfitting, 999 cross-validations in SIMCA-P 13.0 were performed throughout to determine the optimal number of principal components. R^2^X, R^2^Y, and Q^2^Y values of models were nearly 1.0, indicating that these models retain the ability to explain and predict variations in the X and Y matrix.

Furthermore, the value of fold change (FC) was calculated as the average normalized peak intensity ratio between the two groups. Differences between data sets with FC > 1.10 or and *p* < 0.05 (Student *t*-test) were considered statistically significant. The structural identification of differential metabolites was performed by matching the mass spectra with an in-house metabolite library, including accuracy mass, retention time, MS/MS spectra, and online databases Metlin (http://www.metlin.scripps.edu) and HMDB (http://www.hmdb.ca).

The impact of ETEC and GE on metabolic pathways was evaluated based on the MetaboAnalyst platform, a tool for metabolomics data analysis platform, which is available online (https://www.metaboanalyst.ca). The pathway analysis module combines results from powerful pathway enrichment analysis with pathway, topology analysis to help researchers identify the most relevant pathways involved in the conditions being investigated. The analysis report was then presented graphically as well as in a detailed table. Potential biomarkers for GE efficacy were identified based on the metabolic pathway enrichment and statistics analysis.

### Statistical Analysis

Statistical analysis of the integral optical density among the groups was evaluated by using the one-way analysis of variance (ANOVA), performed using SPSS 23.0 (IBM-SPSS Inc., Chicago, USA). The results were presented as mean ± standard error of mean (SEM). Orthogonal polynomial contrasts were used to test for linear and quadratic effects of GE by comparing with the NC group. Significant differences and extremely significant differences were evaluated by Tukey multiple comparisons test at *p* < 0.05 and *p* < 0.01, respectively.

## Results

### Immunohistochemistry

**Figure 1:**
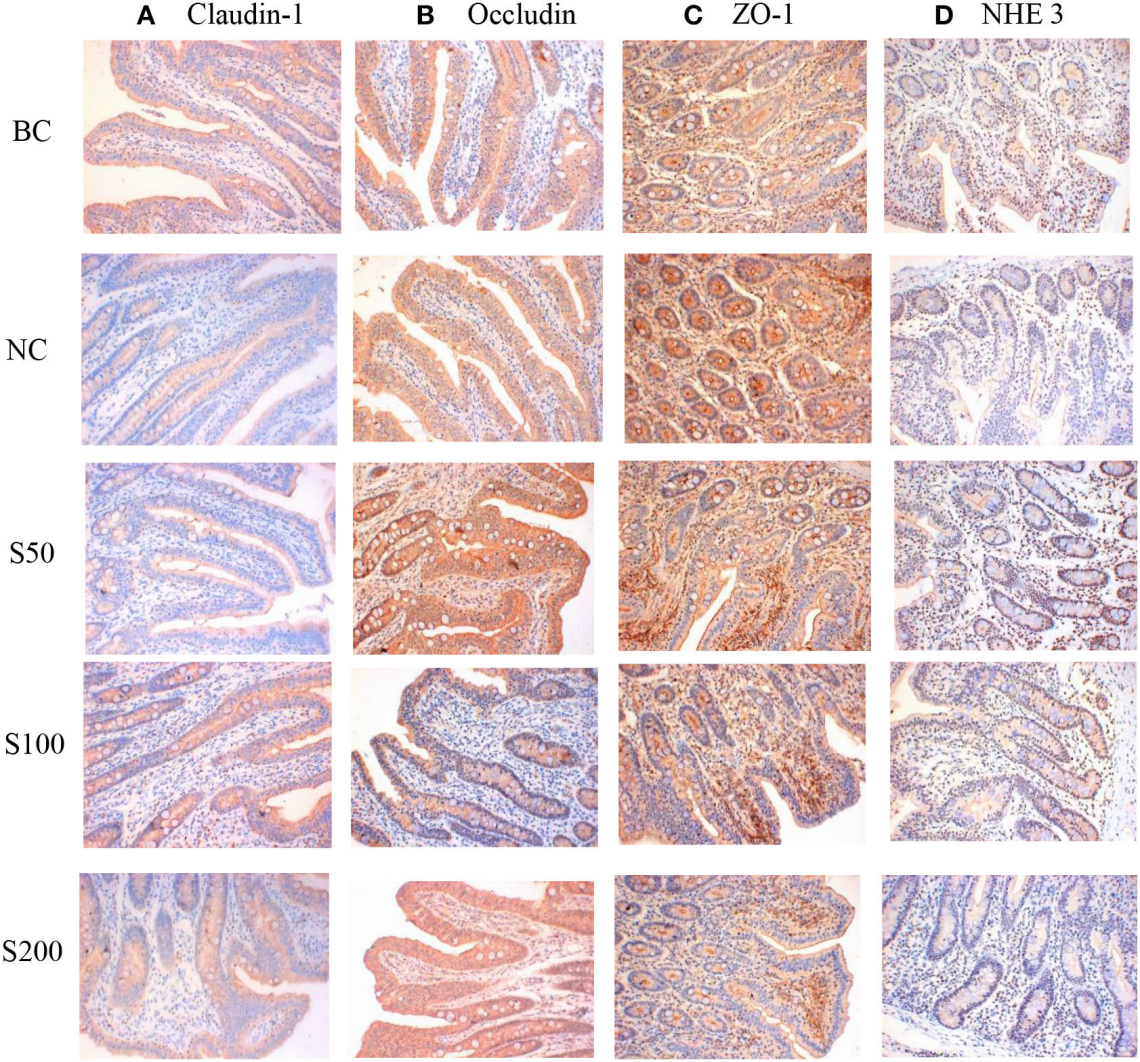
The representative figure of jejunal mucosal claudin-1 **(A)**, occludin **(B)**, zonula occludens 1 (ZO-1) **(C)**, and Na^+^/H^+^ exchanger 3 (NHE3) **(D)** protein expression in different groups (immunohistochemical staining, ×200). The staining was visualized using DAB (brown), and slides were counterstained with hematoxylin (*n* = 5). BC, blank control group, piglets were fed diet without supplements and ETEC challenge; NC, negative control group, piglets were fed diet without supplements and challenged by ETEC; S50, piglets were fed diet supplemented with 50 mg kg^−1^ of GE and challenged by ETEC; S100, piglets were fed diet supplemented with 100 mg kg^−1^ of GE and challenged by ETEC; S200, piglets were fed diet supplemented with 200 mg kg^−1^ of GE and challenged by ETEC.

As seen in Figure 1 and Table 1, the color signals and the integral optical density of occludin and claudin-1 in the S100 and S200 groups were significantly higher than in the NC group (*p* < 0.05), and NHE3 in the GE groups (GEs) was significantly higher than that of the NC group (*p* < 0.05). Supplementation of GE in the diet linearly and quadratic increased the integral optical density of claudin-1 (*p* < 0.01), occludin (*p* < 0.01), and NHE3 (*p* < 0.01) compared with the NC group.

**Table 1:** Effect of guava extract (GE) on the integral optical density of claudin-1, occludin, zonula occludens 1 (ZO-1), and Na^+^/H^+^ exchanger 3 (NHE3) in jejunal mucosa of piglets.

**Items**	**Groups**					**SEM**	***P***	**Contrast^a^**	
	**BC**	**NC**	**S50**	**S100**	**S200**			***L***	***Q***
Claudin-1	22.59^a^	5.98^d^	10.09^cd^	15.05^b^	14.03^bc^	1.53	<0.01	<0.01	<0.01
Occludin	52.08^b^	29.41^c^	55.93^b^	53.62^b^	74.29^a^	3.83	<0.01	<0.01	<0.01
ZO-1	34.49^a^	24.95^b^	29.47^ab^	26.47^b^	25.51^b^	1.03	<0.01	0.574	0.059
NHE3	12.93^a^	3.24^c^	6.60^b^	6.86^b^	6.21^b^	0.86	<0.01	<0.01	<0.01

*In the same row, values with no letter or the same letter superscripts mean no significant difference (p > 0.05), while with different letter superscripts mean significant difference (p < 0.05), n = 5. BC, blank control group, piglets were fed diet without supplements and ETEC challenge; NC, negative control group, piglets were fed diet without supplements and challenged by ETEC; S50, piglets were fed diet supplemented with 50 mg kg^−1^ of GE and challenged by ETEC; S100, piglets were fed diet supplemented with 100 mg kg^−1^ of GE and challenged by ETEC; S200, piglets were fed diet supplemented with 200 mg kg^−1^ of GE and challenged by ETEC*.

a *L, linear; Q, quadratic. Linear and quadratic effect of adding GE compared with the NC*.

### Analysis of Fecal Metabolomics

**Figure 2:**
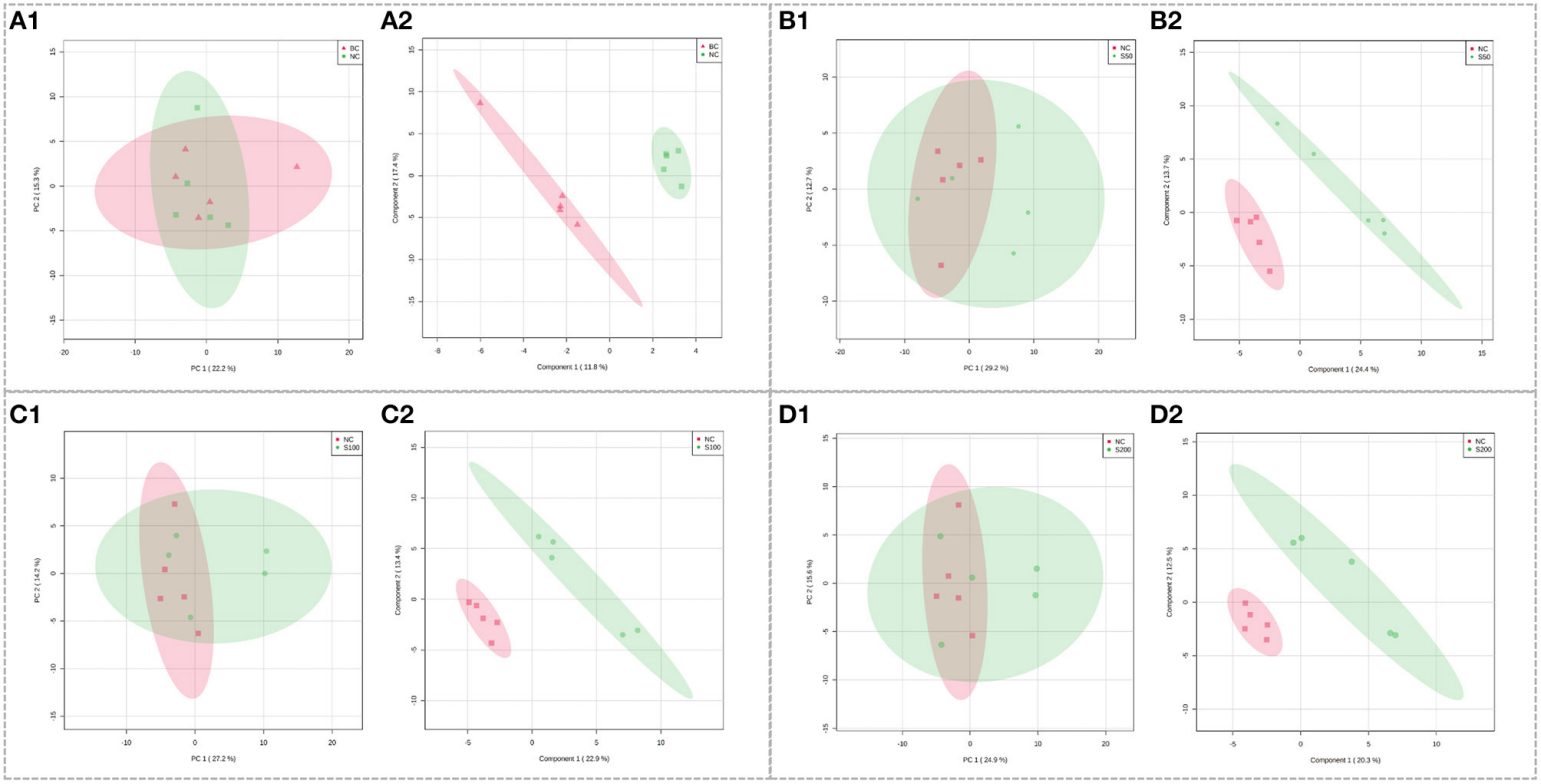
Score plots of principal component analysis (PCA) models in fecal metabolomics. **(A1,B1,C1,D1)** represent the score plots of the PCA models (NC vs. BC group, S50 vs. NC group, S100 vs. NC group, and S200 vs. NC group, respectively), and **(A2,B2,C2,D2)** represent the score plots of the PLS-DA models (NC vs. BC group, S50 vs. NC group, S100 vs. NC group, and S200 vs. NC group, respectively).

Using the optimal UHPLC-QTOF-MS condition described above, the representative total ion chromatograms (TICs) for fecal samples are presented in Supplementary Figure 1A. The score plots of PCA overlapped partly both in the direction of PC1 and PC2 based on the fecal samples from the NC vs. BC group (Figure 2A1), S50 vs. NC group (Figure 2B1), S100 vs. NC group (Figure 2C1), and S200 vs. NC group (Figure 2D1). Supervised PLS-DA analysis suggested that there were significant differences between two groups, indicating the distinct metabolic profiling of the NC vs. BC group (Figure 2A2), S50 vs. NC group (Figure 2B2), S100 vs. NC group (Figure 2C2), and S200 vs. NC group (Figure 2D2).

Metabolic profiling in the feces was significantly changed based on the results of the NC vs. BC group, S50 vs. NC group, S100 vs. NC group, and S200 vs. NC group, respectively (Supplementary Table 2). *L*-pipecolic acid is a unique differential metabolite between the NC group and BC group. Different metabolic pathways were enriched from the groups of S50 vs. NC, S100 vs. NC, and S200 vs. NC, respectively (Supplementary Figure 2). The details of the top 4 ranked metabolic pathways and relevant differential metabolites between the BC, NC, and GEs groups are presented in Table 2. It shows that the S50 group significantly boosted the production of 3-methoxytyramine (*p* < 0.05) and decreased the production of epinephrine, normetanephrine, *N*-acetylserotonin, melatonin, and caffeine (*p* < 0.05) compared with the NC group. The S100 group significantly upregulated the levels of biliverdin and *L*-pipecolic acid (*p* < 0.05), and downregulated the levels of 5-aminolevulinic acid and phosphorylcholine (*p* < 0.05) compared with the NC group. Moreover, the S200 group significantly downregulated the levels of uridine 5′-monophosphate (UMP), deoxycytidine monophosphate (dCMP), deoxyguanosine, and *L*-phenylalanine (*p* < 0.05) compared with the NC group.

**Table 2:** Top 4 ranked metabolic pathways and relevant differential metabolites in the feces of piglets.

**Groups**	**Metabolic pathways**	**Differential metabolites**	
		**Upregulation (*p* < 0.05, FC > 1.10)**	**Downregulation (*p* < 0.05, FC <0.90)**
NC vs. BC	/	/	*L*-pipecolic acid
S50 vs. NC	Tyrosine metabolism	3-methoxytyramine	Epinephrine; normetanephrine
	Tryptophan metabolism	/	*N*-acetylserotonin; melatonin
	Catecholamine biosynthesis	/	Epinephrine
	Caffeine metabolism	/	Caffeine
S100 vs. NC	Porphyrin metabolism	biliverdin	5-Aminolevulinic acid
	Phosphatidylcholine biosynthesis	/	Phosphorylcholine
	Phospholipid biosynthesis	/	Phosphorylcholine
	Lysine degradation	*L*-pipecolic acid	/
S200 vs. NC	Pyrimidine metabolism	/	UMP; dCMP
	Lactose synthesis	/	UMP
	Phenylalanine and tyrosine metabolism	/	*L*-phenylalanine
	Purine metabolism	/	Deoxyguanosine

### Analysis of Serum Metabolomics

**Figure 3:**
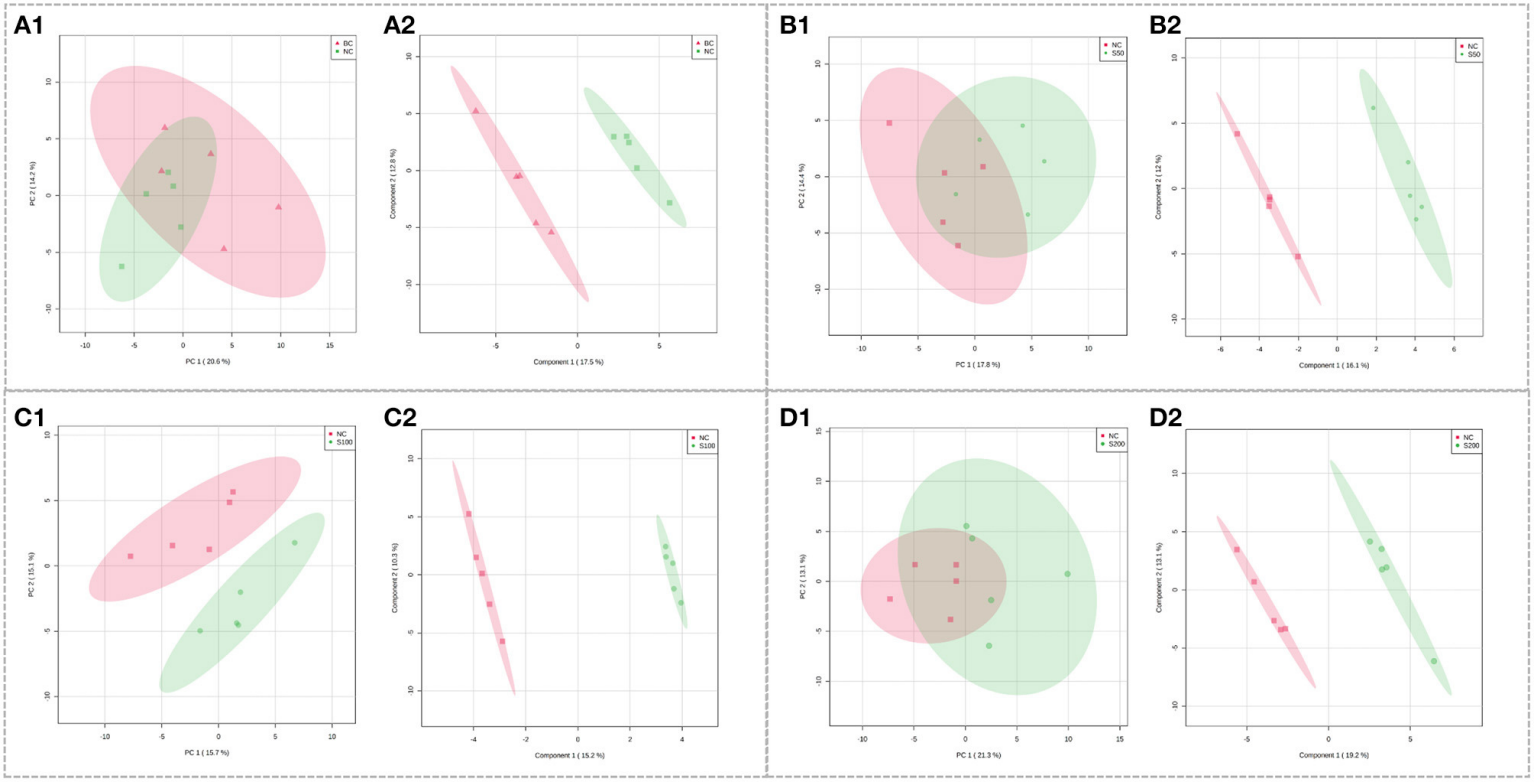
Score plots of PCA models in serum metabolomics. **(A1,B1,C1,D1)** represent the score plots of the PCA models (NC vs. BC group, S50 vs. NC group, S100 vs. NC group, and S200 vs. NC group, respectively), and **(A2,B2,C2,D2)** represent the score plots of the PLS-DA models (NC vs. BC group, S50 vs. NC group, S100 vs. NC group, and S200 vs. NC group, respectively).

The UHPLC-QTOF-MS system can picture metabolic profiling of five groups with TIC (Supplementary Figure 1B). To investigate the global metabolic rewiring in the serum among NC, BC, S50, S100, and S200 groups, all observations were integrated and analyzed using PCA (Figure 3). The score plots of PCA overlapped partly both in the direction of PC1 and PC2 based on the serum samples from the NC vs. BC group (Figure 3A1) and the S50 vs. NC group (Figure 3B1). The score plots of PCA from the S100 vs. NC group (Figure 3C1) were separated from each other. To further explore the differences between the two groups, supervised PLS-DA was applied for chemometrics analysis (Figure 3). The score plots of PLS-DA showed that the NC vs. BC group (Figure 3A2), S50 vs. NC group (Figure 3B2), S100 vs. NC group (Figure 3C2), and S200 vs. NC group (Figure 3D2) could be clearly separated, which reflected the remarkably distinct metabolic status of the serum samples among the BC, NC, S50, S100, and S200 groups. Many metabolites in the serum were significantly altered based on the results of the NC vs. BC group, S50 vs. NC group, S100 vs. NC group, and S200 vs. NC group, respectively (Supplementary Table 3). Finally, different metabolic pathways were identified and further enriched as referred to as the KEGG pathway (Supplementary Figure 3).

**Figure 4:**
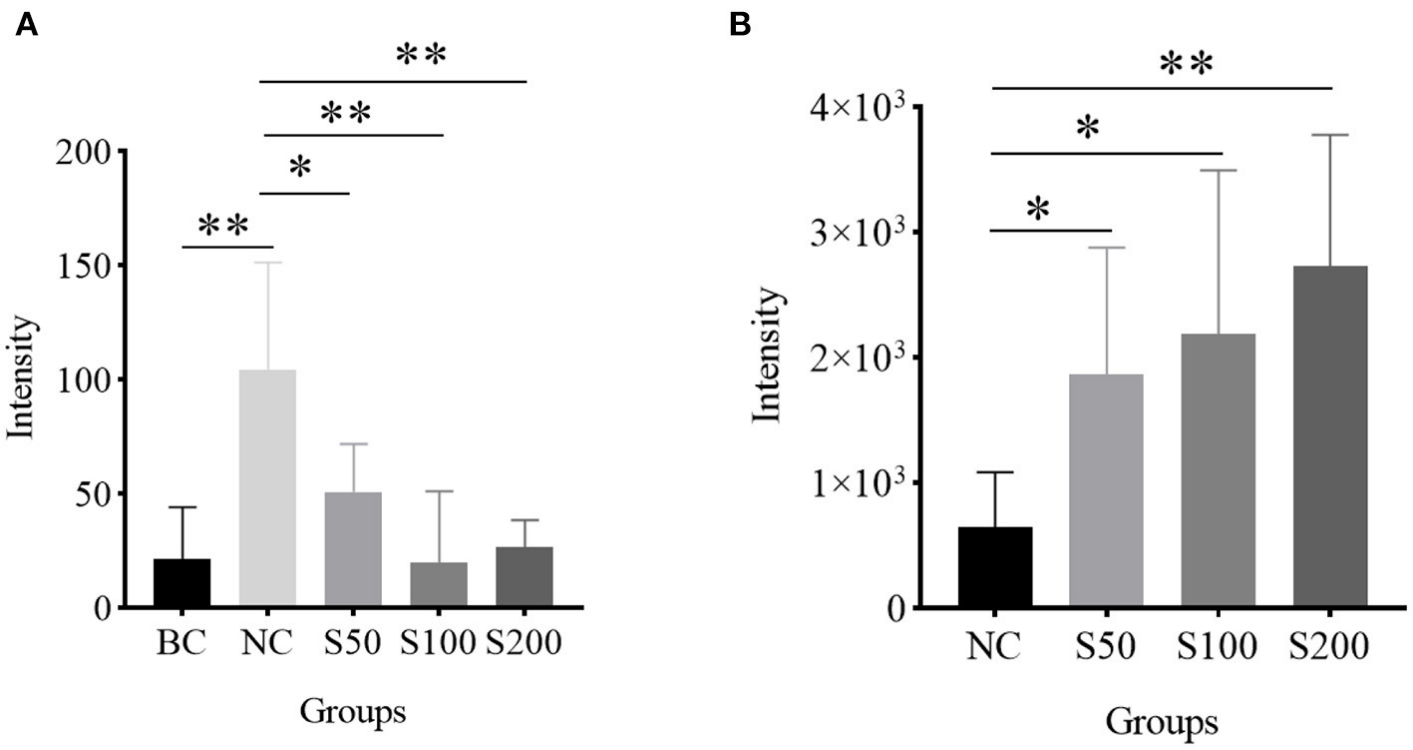
Histogram analysis of key node molecules detected in the serum of piglets: **(A)** nicotinamide-adenine dinucleotide phosphate (NADP); **(B)** tetrahydrofolic acid (THF). *p < 0.05; **p < 0.01.

As shown in Table 3, the top 4 ranked metabolic pathways between the BC, NC, and GE groups displayed characteristic differences in the serum of piglets, respectively. It is interesting that nicotinamide-adenine dinucleotide phosphate (NADP), as a node molecule, was upregulated in the NC group in comparison with the BC group and downregulated in the GE groups in comparison with the NC group (Figure 4A). Notably, tetrahydrofolic acid (THF) is a key node molecule affected by GE, and GE supplementation significantly upregulated the level of THF (*p* < 0.05) compared with the NC group (Figure 4B). Additionally, considering dosage influence on the GE groups, the important top 4 ranked metabolic pathways and relevant metabolites affected also yielded dissimilar results. Especially, it suggested that GE in the S100 group significantly increased the synthesis of adenosine triphosphate (ATP), *L*-glutamic acid (*L*-glu), and *L*-glutamine (Gln) (*p* < 0.05) compared with the NC group. On the other hand, the NC group significantly reduced the production of thiamine pyrophosphate (TPP) (*p* < 0.05) compared with the BC group. However, GE in the S200 group significantly increased the production of TPP and *L*-aspartic acid (*p* < 0.05) compared with the NC group.

**Table 3:** Top 4 ranked metabolic pathways and relevant differential metabolites in the serum of piglets.

**Groups**	**Metabolic pathways**	**Differential metabolites**	
		**Upregulation (*p* < 0.05, FC > 1.10)**	**Downregulation (*p* < 0.05, FC <0.90)**
NC vs. BC	Glutamate metabolism	NADP	Glucosamine 6-phosphate; oxidized glutathione
	Glutathione metabolism	NADP	Oxidized glutathione
	Transfer of acetyl groups into mitochondria	NADP; thiamine pyrophosphate	**/**
	Pyrimidine metabolism	NADP	CMP; dUTP
S50 vs. NC	Glycerolipid metabolism	Glycerol	NADP
	Pterine biosynthesis	Tetrahydrofolic acid	NADP
	Folate metabolism	Tetrahydrofolic acid	NADP
	Histidine metabolism	Tetrahydrofolic acid	NADP
S100 vs. NC	Amino sugar metabolism	*L*-glutamic acid; ATP; *L*-glutamine; glucosamine 6-phosphate	Uridine diphosphate-*N*-acetylglucosamine
	Betaine metabolism	*L*-methionine; tetrahydrofolic acid; ATP; *S*-adenosylhomocysteine	**/**
	Nicotinate and nicotinamide metabolism	*L*-glutamic acid; ATP; *L*-glutamine; *S*-adenosylhomocysteine	NADP
	Glycine and serine metabolism	*S*-adenosylhomocysteine; *L*-glutamic acid; 5-aminolevulinic acid; ATP; *L*-methionine; tetrahydrofolic acid	**/**
S200 vs. NC	Betaine metabolism	*L*-methionine; tetrahydrofolic acid	**/**
	Transfer of acetyl groups into mitochondria	**/**	NADP; thiamine pyrophosphate
	Phytanic acid peroxisomal oxidation	**/**	NADP; thiamine pyrophosphate
	Pterine biosynthesis	Tetrahydrofolic acid	NADP

## Discussion

### Intestinal Mucosal Barrier

In our previous study, the diarrhea incidences of piglets in the BC, NC, S50, S100, and S200 were 1.79, 21.43, 14.29, 8.93, and 7.14%, respectively. It suggested that dietary addition of GE could reduce diarrhea incidence significantly in weaned piglets challenged by ETEC (17). In general, diarrhea disease caused by ETEC infections is a major risk factor for impaired intestinal structure and barrier function of piglets. It has been reported that claudins and occludins are considered in the tight junction protein components, which primarily regulated the permeability of uncharged and charged molecules. Furthermore, ZO-1 is the adaptor protein that modulates the actin cytoskeleton (24, 25), and NHE3 is a primary mediator of the absorptive route for Na^+^ entering the intestinal epithelium from the lumen (26). Thus, all of them play important roles in mediating the functional integrity of the junction in epithelial and endothelial cells of the intestines (24–26). Our results showed that ETEC decreased the expression of epithelial tight junctions, such as claudin-1, occludin, ZO-1, and NHE3, thereby, in turn, increasing cellular permeability and disturbed the intestinal mucosal barrier. Subsequently, luminal antigens rather than bacteria may enter the lamina propria, resulting in inflammation (27). However, weaned piglets fed a diet supplementation with GE (50–200 mg kg^−1^ in the diet) are characterized by increased expression of claudin-1, occludin, ZO-1, and NHE3, which are crucial for the formation of a semipermeable mucosal barrier and the recovery of the barrier function of intestinal tight junctions compared with the NC group. Furthermore, previous studies also suggested that GE was rich in phenolics (28), and phenolics have a positive effect on gut health (29). Specifically, quercetin and myricetin, known as the main phenolic constituents in GE (17, 30), have been demonstrated to enhance intestinal barrier function (31). Consequently, it is assumed that the abundant phenolics in GE exerted anti-inflammatory (32) and anti-diarrhea activity (15), and improved the intestinal barrier function and gut mucosal integrity of piglets.

### Fecal Metabolomics

**Figure 5:**
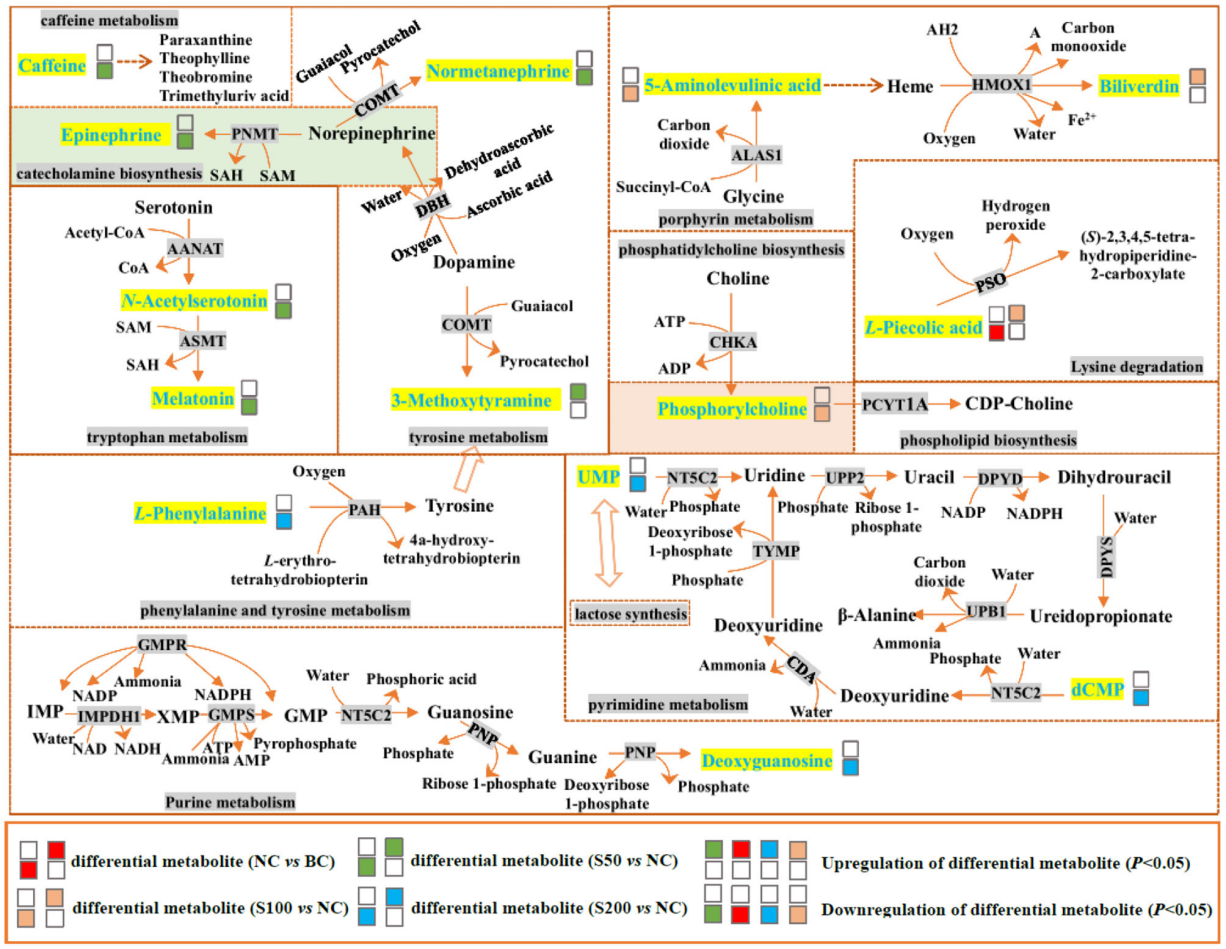
Top 4 ranked metabolic pathways and related differential metabolites in the feces of piglets. The details of abbreviated metabolites: AANAT, serotonin *N*-acetyltransferase; ADP, adenosine diphosphate; AMP, adenosine monophosphate; ASMT, acetylserotonin *O*-methyltransferase; ATP, adenosine triphosphate; ALAS1, 5-aminolevulinate synthase; CDA, calcium-transporting ATPase; CHKA, choline kinase alpha; COMT, catechol *O*-methyltransferase; DBH, dopamine beta-hydroxylase; DPYD, dihydropyrimidine dehydrogenase [NADP(+)]; DPYS, dihydropyrimidinase; GMP, guanosine 5′-monophosphate; GMPR, guanosine 5′-monophosphate oxidoreductase 1; GMPS, GMP synthase (glutamine hydrolyzing); HMOX1, heme oxygenase; IMP, inosine-5′-monophosphate; IMPDH1, inosine-5′-monophosphate dehydrogenase 1; NAD, nicotinamide adenine dinucleotide; NADH, reduced nicotinamide adenine dinucleotide; NADP, nicotinamide adenine dinucleotide phosphate; NADPH, reduced nicotinamide adenine dinucleotide phosphate; NT5C2, cytosolic purine 5′-nucleotidase; PAH, phenylalanine-4-hydroxylase; PCYT1A, choline phosphate cytidylyltransferase A; PNMT, phenylethanolamine *N*-methyltransferase; PNP, polyribonucleotide nucleotidyltransferase; PSO, peroxisomal sarcosine oxidase; SAH, *S*-adenosyl-*L*-homocysteine; SAM, *S*-adenosylmethionine; TYMP, thymidine phosphorylase; UPB1, beta-ureidopropionase; UPP2, uridine phosphorylase 2; XMP, xanthosine monophosphate; AH2 and A are two generic compounds.

In mammals, *L*-pipecolic acid has long been recognized as a metabolite of lysine degradation (33). In this pathway, peroxisomal sarcosine oxidase (PSO) can catalyze *L*-pipecolic acid and oxygen to yield (*S*)-2,3,4,5-tetrahydropiperidine-2-carboxylate and hydrogen peroxide (H_2_O_2_). As seen in Figure 5 and Table 2, it indicated that *L*-pipecolic acid was significantly lower in the NC compared with the BC group and suggested that ETEC might have activated the reactions mentioned above, and led to the consumption of *L*-pipecolic acid and the production of H_2_O_2_. H_2_O_2_ accumulation can induce disruption of the intestinal epithelial barrier function by a mechanism involving phosphatidylinositol 3-kinase and c-Src kinase (34, 35). Here, consistent with the results of immunohistochemistry, in the present study, it suggests that H_2_O_2_-induced oxidative stress in the gut might have been considered to be one of the important pathogenic mechanisms in the NC compared with the BC group, which disrupted intestinal epithelial tight junctions and barrier functions.

In addition, catecholamines are generally associated with stress events that result in high levels of Gram-negative pathogens, such as *Escherichia coli* (36). The metabolic results between the S50 vs. NC group suggested that dietary addition with 50 mg kg^−1^ GE can decrease the production of stress hormones, such as catecholamines (epinephrine and normetanephrine), and increase the production of 3-methoxytyramine (an inactive metabolite of dopamine) through tyrosine metabolism and catecholamine biosynthesis pathways and finally inhibit the growth of *Escherichia coli* against oxidative stress in the gut. In addition, it has been reported that caffeine can increase intracellular calcium levels through direct effects on metabolic phosphorylase-like enzyme (PHOS) regulation and calcium mobilization from the sarcoplasmic reticulum (37). Compared with the NC group, the caffeine level in the feces from the S50 group was downregulated, which may reduce the intracellular calcium content, thereby inhibiting the pathophysiological process that leads to diarrhea (38).

**Figure 6:**
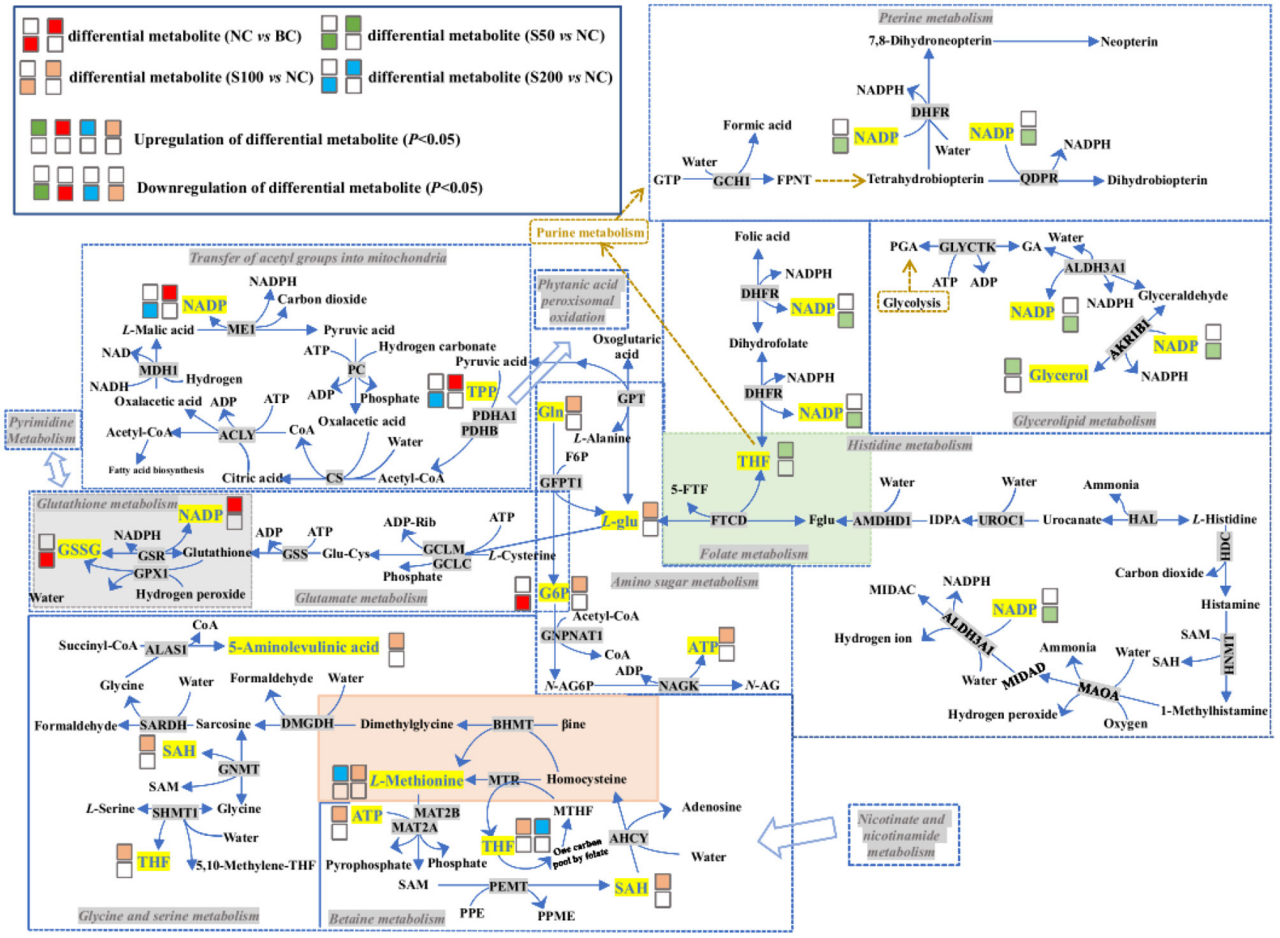
Top 4 ranked metabolic pathways and related differential metabolites in the serum of piglets. The details of abbreviated metabolites: ACLY, ATP-citrate synthase; ADP-Rib, adenosine diphosphate ribose; AHCY, adenosylhomocysteinase; AKR1B1, aldose reductase; ALAS1, 5-aminolevulinate synthase; ALDH3A1, aldehyde dehydrogenase dimeric NADP-preferring; AMDHD1, probable imidazolonepropionase; BHMT, betaine–homocysteine S-methyltransferase 1; βine, betaine; CS, citrate synthase; DHFR, dihydrofolate reductase; DMGDH, dimethylglycine dehydrogenase, mitochondrial; F6P, fructose 6-phosphate; Fglu, N-formyl-*L*-glutamic acid; FPNT, formamidopyrimidine nucleoside triphosphate; 5-FTF, N5-formyl-THF; FTCD, formimidoyltransferase-cyclodeaminase; G6P, glucosamine 6-phosphate; GA, glyceric acid; GCH1, GTP cyclohydrolase 1; GCLC, glutamate–cysteine ligase catalytic subunit; GCLM, glutamate–cysteine ligase regulatory subunit; GFPT1, glutamine-fructose-6-phosphate aminotransferase; Gln, L-glutamine; Glu-Cys, glutamylcycteine; GLYCTK, glycerate kinase; GNMT, glycine N-methyltransferase; GNPNAT1, glucosamine 6-phosphate N-acetyltransferase; GPT, glutamate pyruvate transaminase; GPX1, glutathione peroxidase 1; GSSG, oxidized glutathione; GSR, glutathione reductase; GSS, glutathione synthetase; GTP, guanosine triphosphate; HAL, histidine ammonia-lyase; HDC, histidine decarboxylase; HNMT, histamine N-methyltransferase; IDPA, 4-imidazolone-5-propionic acid; L-glu, *L*-glutamic acid; MAOA, amine oxidase (Flavin containing) A; MAT2A, *S*-adenosylmethionine synthase isoform type-2; MAT2B, methionine adenosyltransferase 2 subunit beta; MDH1, malate dehydrogenase; ME1, NADP-dependent malic enzyme; MIDAC, methylimidazoleacetic acid; MIDAD, methylimidazole acetaldehyde; MTHF, 5-methyltetrahydrofolic acid; MTR, methionine synthase; N-AG6P, N-acetyl-D-glucosamine-6-phosphate; N-AG, N-acetyl-*D*-glucosamine; NAGK, N-acetyl-D-glucosamine kinase; PC, pyruvate carboxylase; PDHA1, pyruvate dehydrogenase E1-alpha; PDHB, pyruvate dehydrogenase E1-beta; PEMT, phosphatidylethanolamine N-methyltransferase; PGA, 3-phosphoglyceric acid; PPE, phosphatidyl-ethanolamide; PPME, phosphatidyl-N-methylethanolamide; QDPR, dihydropteridine reductase; SARDH, sarcosine dehydrogenase; SHMT1, serine hydroxymethyltransferase cytosolic; THF, tetrahydrofolic acid; TPP, thiamine pyrophosphate; UROC1, urocanate hydratase.

Based on the fecal metabolomics data of the S100 vs. NC group, it showed that 100 mg kg^−1^ of GE upregulated the level of biliverdin and downregulated the level of 5-aminolevulinic acid in the gut via the porphyrin metabolism pathway. This process started as the condensation of glycine and succinyl-CoA by 5-aminolevulinate synthase (ALAS) and generated 5-aminolevulinic acid. Presumably, the resulting 5-aminolevulinic acid has two fates. On one hand, it may be finally converted into biliverdin via a series of metabolic steps, leading to the accumulation of biliverdin in the gut (Figure 5). The biliverdin generated in this process can protect the intestines from oxidants and inflammation (39, 40). On the other hand, 5-aminolevulinic acid was also potentially absorbed into the blood, resulting in the high level of 5-aminolevulinic acid in the serum via glycine and serine metabolism (Figure 6). Here, 5-aminolevulinic acid reduced intracellular carbon monoxide and inhibited oxidative stress and inflammation response (41). Moreover, phosphorylcholine was downregulated in the S100 group compared with the NC group, which was associated with the phosphatidylcholine and phospholipid biosynthesis pathways. It means that most of the choline of the S100 group might not be catabolized in the gut but was absorbed into the blood, and then it fluxed into betaine metabolism and was probably utilized for betaine biosynthesis (Figure 6). Notably, *L*-pipecolic acid in the feces of the S100 group was significantly higher than that of the NC group. Conversely, *L*-pipecolic acid in the feces of the NC group was significantly lower than that of the BC group. Our findings suggested that 100 mg kg^−1^ of GE might inhibit the activity of PSO and reverse the lower levels of *L*-pipecolic acid caused by ETEC, which in turn prevented the production of H_2_O_2_ and decreased oxidative stress level.

Based on the S200 vs. NC group, *L*-phenylalanine was downregulated, which is a double-edged sword. First, *L*-phenylalanine is not only an essential amino acid but also a precursor of tyrosine and catecholamines (including tyramine, dopamine, epinephrine, and norepinephrine), so the lower level of *L*-phenylalanine might decrease oxidative stress in the intestine (36). Second, the lower level of *L*-phenylalanine also might lead to a decrease in gut hormone secretion, including glucose-dependent insulinotropic peptide (GIP) and cholecystokinin (CCK) (42). GIP and CCK are important hormonal regulators of the ingestion, digestion, and absorption of intestinal nutrients (43, 44). Additionally, our results indicated that UMP and dCMP were downregulated in the S200 group compared with the NC group. Both of them were involved in the pyrimidine metabolism and lactose synthesis pathways and suggested that UMP and dCMP finally may be degraded to β-alanine through the pyrimidine metabolism pathway, and then the β-alanine synthesized probably fluxed into the alanine metabolism pathway. In this pathway, alanine and glyoxylic acid can be converted into glycine and pyruvic acid via serine-pyruvate aminotransferase. Meanwhile, *D*-glucose probably participated in the biosynthesis of pyruvic acid, leading to the lower level of UMP in the lactose synthesis pathway. Then the pyruvic acid generated via two pathways may be absorbed into the blood and was involved in the transfer of acetyl groups into the mitochondria pathway (Figure 6). Furthermore, our data revealed that the lower level of deoxyguanosine in the feces was associated with the higher level of inosine-5′-monophosphate (IMP) in the serum in the S200 group compared with the NC group (Supplementary Table 3), while the higher level of IMP, as a nucleotide, may be propitious to the growth and maturation of intestinal epithelial cells and plays an important role in intestinal immunity and health (45).

### Serum Metabolomics

As seen in Figure 6 and Table 3, based on the NC vs. BC group, ETEC challenge decreases glucosamine 6-phosphate (G6P) and oxidized glutathione (GSSG) levels and increases NADP levels in the serum by affecting the glutamate and glutathione metabolism pathways, which may result in the accumulation of H_2_O_2_ in the serum. On one hand, the produced H_2_O_2_ cannot be reduced to water (H_2_O), which resulted in peroxide interference and cell damage through oxidation of lipids, proteins, and nucleic acids (46). On the other hand, H_2_O_2_ is not a radical but is considered a reactive oxygen species, which can induce a cascade of radical reactions and inactivate pyruvate dehydrogenase (PDH) (47, 48), leading to accumulation of TPP in the serum and meaning that pyruvic acid cannot be synthesized into acetyl-CoA, while the latter was closely associated with fatty acid biosynthesis. In addition, the lower levels of cytidine monophosphate (CMP) and deoxyuridine triphosphate (dUTP) in the NC group compared with the BC group revealed that ETEC perturbed pyrimidine metabolism, and then, it might inhibit the process of pyrimidine-related nucleotide biosynthesis.

Interestingly, based on the S50 vs. NC group, caffeine was significantly downregulated in the feces (Table 2), while it was significantly upregulated in the serum (Supplementary Table 3) and suggested that most caffeine can be absorbed into the blood through the intestinal mucosa. Furthermore, the high level of caffeine in the serum could lead to an increase in lipolysis, and it is usually accompanied by the accumulation of glycerol in the serum (49). Then the high level of glycerol could raise blood osmolality, and it, in turn, probably plays a favorable role in the increase in intestinal water absorption and the decrease in sodium efflux into the intestinal lumen, and finally resulted in the attenuation of secretory diarrhea caused by ETEC (50). It is worth noting that, based on the S50 vs. NC group, indoleamine 2,3-dioxygenase 1 (IDO1) or tryptophan 2,3-dioxygenase 2 (TDO2) drives tryptophan into the kynurenine pathways that produce tryptophan catabolites, such as the high level of kynurenic acid in the serum (Supplementary Table 3). In this process, it is usually accompanied by the production of folic acid and *L*-glu, meaning that the generated folic acid and *L*-glu can be synthesized to THF through the folate metabolism pathway. Meanwhile, THF also was biosynthesized in the serum via two pathways, including pterine biosynthesis and histidine metabolism.

Based on the S100 vs. NC group, the higher level of *L*-glu supplies the amino group for the biosynthesis of other amino acids, is a substrate for glutamine and glutathione synthesis, and is the key neurotransmitter in biological systems. It revealed that after glutamine synthetase or glutaminase liver isoform (GLS2) converts *L*-glu into Gln, and glutamine-fructose-6-phosphate aminotransferase (GFPT1) subsequently converts Gln and fructose 6-phosphate (F6P) into *L*-glu and G6P, it suggested that 100 mg kg^−1^ of GE reversed the ETEC-induced downregulation of G6P. The higher level of G6P in the S100 group, in turn, can be converted into *N*-acetyl-*D*-glucosamine-6-phosphate (*N*-AG6P) (via glucosamine 6-phosphate *N*-acetyltransferase) compared with the NC group. Here, downregulation of uridine diphosphate-*N*-acetylglucosamine and upregulation of ATP in serum indicated that most *N*-AG6P generated likely can be converted into *N*-acetyl-*D*-glucosamine (*N*-AG) (a polysacchatide) and ATP, via *N*-acetyl-*D*-glucosamine kinase (NAGK). The resulting *N*-AG has confirmed its anti-inflammatory efficacy for inflammatory bowel disease (51). It is worth mentioning that betaine, which might be synthesized from choline, can be degraded via two pathways. The first pathway involves betaine metabolism. Compared with the NC group, the higher levels of *S*-adenosyl-*L*-homocysteine (SAH), *L*-methionine, THF, and ATP in the S100 group indicated that THF cofactors were probably used to carry and activate one-carbon units via the folate-mediated one-carbon transfer pathway, resulting in the remethylation of homocysteine to *L*-methionine, and the synthesis of purine nucleotides and thymidylate (52). In the second pathway, betaine can be synthesized to dimethylglycine in the methionine cycle, then the generated dimethylglycine can be converted into sarcosine and enter glycine and serine metabolism. Here, the sarcosine is synthesized via two pathways to create 5-aminolevulinic acid and serine, respectively. Of them, the formation pathway of serine was accompanied by the production of THF, whereas the production of *L*-methionine, purine nucleotides, and 5-aminolevulinic acid may participate in the processes of attenuated inflammatory responses and inhibited oxidative stress (53–55).

Furthermore, it showed that 200 mg kg^−1^ of GE reversed the ETEC-induced upregulation of NADP and TPP in the serum via the transfer of acetyl groups into the mitochondria and phytanic acid peroxisomal oxidation pathways and thereby participated in the production of acetyl-CoA, and the latter was related to the synthesis of fatty acids and sterols and the metabolism of many amino acids (56). Meanwhile, similar to the S50 or S100 groups, the levels of THF and *L*-methionine in the S200 group were also upregulated via betaine metabolism and the pterine biosynthesis pathway compared with the NC group.

It is worth noting that GE dietary addition can upregulate the level of THF and reverse the high level of NADP induced by ETEC compared with the NC group (Figure 4). It suggested that THF is probably a main antioxidative force for GE indirectly (57, 58). Meanwhile, GE downregulating the level of NADP also means that the NADP pool is probably maintained in a highly reduced state, which boosted antioxidant ability in response to oxidative damage (59).

## Conclusions

Our study has demonstrated that dietary supplementation with 50–200 mg kg^−1^ of GE exhibited a positive effect on the recovery of intestinal tight junctions and barrier function of weaned piglets challenged by ETEC. Meanwhile, serum and fecal metabolomics analysis indicated that dietary GE (50, 100, and 200 mg kg^−1^) addition could reprogram energy metabolism through similar or distinct metabolic pathways and finally enhance the antioxidant ability of weaned piglets challenged by ETEC.

## Data Availability Statement

The raw data supporting the conclusions of this article will be made available by the authors without undue reservation.

## Ethics Statement

The animal study was reviewed and approved by Institutional Animal Care and Use Committee of the Chinese Academy of Tropical Agricultural Sciences (Haikou, China).

## Author Contributions

DW and HZ contributed to the study design. LZ analyzed the data and wrote the manuscript. DW and GH finished the animal experiments and determination. All authors reviewed and approved the final version of the manuscript.

## Conflict of Interest

The authors declare that the research was conducted in the absence of any commercial or financial relationships that could be construed as a potential conflict of interest.

## Abbreviations

ALAS: 5-aminolevulinate synthase
ATP: adenosine triphosphate
ACN: acetonitrile
CCK: cholecystokinin
CMP: cytidine monophosphate
dCMP: deoxycytidine monophosphate
dUTP: deoxyuridine triphosphate
ESI: electrospray ionization
ETEC: enterotoxigenic *Escherichia coli*
F6P: fructose 6-phosphate
G6P: glucosamine 6-phosphate
GE: guava leaf extract
GFPT1: glutamine-fructose-6-phosphate aminotransferase
GIP: glucose-dependent insulinotropic peptide
Gln: *L*-glutamine
GLS2: glutaminase liver isoform
GSSG: oxidized glutathione
H_2_O_2_: hydrogen peroxide
IDO1: indoleamine 2, 3-dioxygenase 1
IMP: inosine-5′-monophosphate
*L*-glu: *L*-glutamic acid
NADP: nicotinamide-adenine dinucleotide phosphate
*N*-AG6P: *N*-acetyl-*D*-glucosamine-6-phosphate
NHE3: Na^+^/H^+^ exchanger 3
PBS: phosphate-buffered saline
PCA: principal components analysis
PDH: pyruvate dehydrogenase
PHOS: phosphorylase-like enzymes
PLS-DA: partial least-squares discrimination analysis
PSO: peroxisomal sarcosine oxidase
SAH: *S*-adenosyl-*L*-homocysteine
TDO2: tryptophan 2,3-dioxygenase 2
THF: tetrahydrofolic acid
TICs: total ion chromatograms
TPP: thiamine pyrophosphate
UMP: uridine 5′-monophosphate
UHPLC-QTOF-MS: ultra-high-performance liquid chromatography/quadrupole time-of-flight mass spectrometry
ZO-1: zonula occludens protein 1.
