# Supplementary material for: Effects of Guava (Psidium guajava L.) Leaf Extract on the Metabolomics of Serum and Feces in Weaned Piglets Challenged by Escherichia coli
Source: Front Vet Sci. 2021 May 24;8:656179. doi: 10.3389/fvets.2021.656179 (PMC8183609; doi:10.3389/fvets.2021.656179)
Supplement: Supplementary file 1 [file Data_Sheet_1.docx]

**Table 1S** Composition and nutrient levels of diets (as-fed basis)

| **Ingredient, g kg^-1^** | | **Nutrient levels, g kg^-1^ ^c^** | |
| --- | --- | --- | --- |
| Corn | 579.0 | DE, MJ kg^-1^ | 14.02 |
| Soybean meal | 254.7 | Crude protein | 200.5 |
| Fish meal | 50.0 | Ca | 6.2 |
| Whey powder | 40.0 | Available P | 5.0 |
| Cream powder | 45.0 | Lys | 15.9 |
| Limestone | 3.0 | Met | 5.6 |
| Ca (H_2_PO_4_)_2_ | 12.0 | Met+Cys | 8.5 |
| Moldproofant | 1.0 |  |  |
| Acidifier | 3.0 |  |  |
| L-Lysiine‧HCl | 2.5 |  |  |
| Choline chloride | 1.0 |  |  |
| DL-Methionine | 0.5 |  |  |
| NaCl | 3.0 |  |  |
| Trace mineral premix ^a^ | 5.0 |  |  |
| Vitamin premix ^b^ | 0.3 |  |  |
| Total | 1000.0 |  |  |

^a^ Trace mineral premix provides the following per kilogram of diets: 16.5 mg of Cu as CuSO_4_‧5H_2_O, 100 mg of Fe as FeSO_4_‧H_2_O, 35 mg of Mn as MnSO_4_‧H_2_O, 100 mg of Zn as ZnSO_4_‧H_2_O, 0.3 mg of Se as Na_2_SeO_3_, 0.3 mg of I as KI.

^b^ Vitamin premix provides the following per kilogram of diets: 11000 IU of vitamin A, 1500 IU of vitamin D3, 16 IU of vitamin E，1 mg of vitamin K, 0.3 mg of folic acid, 15 mg of nicotinic, 10 mg of pantothenate, 2.5 mg of biotin, 1 mg of vitamin B1, 4.0 mg of vitamin B2, 0.01 mg of vitamin B12.

^C^ Nutrient levels were calculated values.

**Table 2S** Differential metabolites identified among BC, NC, S50, S100 and S200 groups from the data set of the fecal samples

| **Groups** | **Num.** | **Name** | ***P* value** | **FC** |
| --- | --- | --- | --- | --- |
| **NC *vs* BC** | 1 | *L*-pipecolic acid | 2.51×10^-2^ | 0.34 |
| **S50 *vs* NC** | 1 | tartaric acid | 3.02×10^-3^ | 3.03 |
|  | 2 | *N*-acetylserotonin | 3.47×10^-3^ | 0.61 |
|  | 3 | melatonin | 1.14×10^-2^ | 0.56 |
|  | 4 | 4-hydroxyproline | 1.17×10^-2^ | 0.49 |
|  | 5 | 5-aminolevulinic acid | 1.17×10^-2^ | 0.49 |
|  | 6 | 4-guanidinobutyric acid | 2.29×10^-2^ | 0.61 |
|  | 7 | caffeine | 2.32×10^-2^ | 0.52 |
|  | 8 | normetanephrine | 3.10×10^-2^ | 0.55 |
|  | 9 | epinephrine | 3.10×10^-2^ | 0.55 |
|  | 10 | dCMP | 3.94×10^-2^ | 0.35 |
|  | 11 | 1,3-diaminopropane | 4.07×10^-2^ | 2.54 |
|  | 12 | 3-methoxytyramine | 4.37×10^-2^ | 2.58 |
|  | 13 | 1*H*-indole-3-acetamide | 4.54×10^-2^ | 0.47 |
| **S100 *vs* NC** | 1 | tartaric acid | 3.65×10^-3^ | 1.98 |
|  | 2 | *D*- maltose | 3.89×10^-3^ | 2.28 |
|  | 3 | deoxyguanosine | 1.18×10^-2^ | 0.43 |
|  | 4 | 4-hydroxyproline | 1.90×10^-2^ | 0.38 |
|  | 5 | 5-aminolevulinic acid | 1.90×10^-2^ | 0.38 |
|  | 6 | phosphorylcholine | 2.07×10^-2^ | 0.31 |
|  | 7 | allantoin | 2.31×10^-2^ | 7.25 |
|  | 8 | phosphonoacetate | 2.32×10^-2^ | 2.47 |
|  | 9 | biliverdin | 3.51×10^-2^ | 2.03 |
|  | 10 | glycolate | 4.27×10^-2^ | 1.41 |
|  | 11 | *L*-pipecolic acid | 4.55×10^-2^ | 4.04 |
|  | 12 | quinic acid | 4.62×10^-2^ | 5.15 |
| **S200 *vs* NC** | 1 | tartaric acid | 1.42×10^-3^ | 2.56 |
|  | 2 | *L*-phenylalanine | 1.79×10^-2^ | 0.57 |
|  | 3 | *L*-homocystine | 2.16×10^-2^ | 5.21 |
|  | 4 | dCMP | 2.84×10^-2^ | 0.36 |
|  | 5 | deoxyguanosine | 2.91×10^-2^ | 0.52 |
|  | 6 | UMP (uridine 5'-monophosphate) | 3.52×10^-2^ | 0.69 |
|  | 7 | cyclic GMP | 3.79×10^-2^ | 0.49 |

**Table 3S** Differential metabolites identified among BC, NC, S50, S100 and S200 groups from the data set of the serum samples

| **Groups** | **Num.** | **Name** | ***P* value** | **FC** |
| --- | --- | --- | --- | --- |
| **NC *vs* BC** | 1 | CMP (cytidine monophosphate) | 3.98×10^-2^ | 0.31 |
|  | 2 | dUTP (deoxyuridine triphosphate) | 2.83×10^-2^ | 0.33 |
|  | 3 | 1-methyladenosine | 3.41×10^-2^ | 0.34 |
|  | 4 | *L*-allothreonine | 4.14×10^-2^ | 0.47 |
|  | 5 | GSSG (oxidized glutathione) | 1.44×10^-2^ | 0.51 |
|  | 6 | *N*, *N*-dimethylaniline | 4.02×10^-2^ | 0.51 |
|  | 7 | hydroxykynurenine | 4.43×10^-2^ | 0.54 |
|  | 8 | glucosamine 6-phosphate | 2.71×10^-2^ | 0.83 |
|  | 9 | ITP | 2.89×10^-2^ | 2.00 |
|  | 10 | chenodeoxycholic acid glycine conjugate | 2.44×10^-2^ | 2.11 |
|  | 11 | *L*-kynurenine | 4.65×10^-2^ | 2.28 |
|  | 12 | diaminopimelic acid | 8.46×10^-3^ | 3.64 |
|  | 13 | NADP | 7.22×10^-3^ | 4.89 |
|  | 14 | thiamine pyrophosphate | 4.25×10^-2^ | 6.91 |
| **S50 *vs* NC** | 1 | desthiobiotin | 1.23×10^-2^ | 0.26 |
|  | 2 | 2-phosphoglyceric acid | 2.12×10^-2^ | 0.30 |
|  | 3 | pterin | 3.80×10^-4^ | 0.40 |
|  | 4 | NADP | 4.72×10^-2^ | 0.49 |
|  | 5 | caffeine | 4.76×10^-2^ | 1.85 |
|  | 6 | hypotaurine | 2.87×10^-2^ | 1.87 |
|  | 7 | tryptophanamide | 4.58×10^-2^ | 1.93 |
|  | 8 | glycerol | 1.94×10^-2^ | 2.05 |
|  | 9 | quinic acid | 4.79×10^-3^ | 2.14 |
|  | 10 | 5-aminolevulinic acid | 3.39×10^-2^ | 2.21 |
|  | 11 | *N*-acetyl-*L*-alanine | 3.39×10^-2^ | 2.21 |
|  | 12 | 4-hydroxyproline | 3.39×10^-2^ | 2.21 |
|  | 13 | tetrahydrofolic acid | 3.86×10^-2^ | 2.88 |
|  | 14 | kynurenic acid | 2.54×10^-2^ | 42.12 |
| **S100 *vs* NC** | 1 | phosphocreatine | 4.14×10^-2^ | 0.14 |
|  | 2 | 2,5-dimethylpyrazine | 1.20×10^-2^ | 0.18 |
|  | 3 | NADP | 9.85×10^-3^ | 0.19 |
|  | 4 | desthiobiotin | 1.81×10^-2^ | 0.28 |
|  | 5 | uridine 5'-diphospho-*N*-acetylgalactosamine | 3.66×10^-2^ | 0.37 |
|  | 6 | uridine diphosphate-*N*-acetylglucosamine | 3.66×10^-2^ | 0.37 |
|  | 7 | *N*-acetyltryptophan | 4.09×10^-2^ | 0.44 |
|  | 8 | *L*-glutamic acid | 2.97×10^-2^ | 1.33 |
|  | 9 | *N*-methyl-*D*-aspartic acid | 2.97×10^-2^ | 1.33 |
|  | 10 | *O*-acetylserine | 2.97×10^-2^ | 1.33 |
|  | 11 | 3-sulfinoalanine | 2.74×10^-2^ | 1.43 |
|  | 12 | *L*-glutamine | 5.85×10^-3^ | 1.61 |
|  | 13 | 5-aminolevulinic acid | 2.34×10^-3^ | 1.63 |
|  | 14 | *N*-acetyl-*L*-alanine | 2.34×10^-3^ | 1.63 |
|  | 15 | 4-hydroxyproline | 2.34×10^-3^ | 1.63 |
|  | 16 | glucosamine 6-phosphate | 2.49×10^-2^ | 1.81 |
|  | 17 | salicylamide | 1.77×10^-2^ | 1.89 |
|  | 18 | tryptophanamide | 1.03×10^-2^ | 1.91 |
|  | 19 | *L*-methionine | 3.82×10^-2^ | 2.23 |
|  | 20 | *S*-adenosylhomocysteine | 2.38×10^-3^ | 2.33 |
|  | 21 | CMP (cytidine monophosphate) | 1.34×10^-2^ | 2.84 |
|  | 22 | glycerol | 2.35×10^-2^ | 2.92 |
|  | 23 | ATP (adenosine triphosphate) | 1.49×10^-2^ | 2.97 |
|  | 24 | tetrahydrofolic acid | 3.60×10^-2^ | 3.39 |
|  | 25 | thyrotropin releasing hormone | 5.00×10^-4^ | 10.32 |
| **S200 *vs* NC** | 1 | thiamine pyrophosphate | 3.39×10^-2^ | 0.10 |
|  | 2 | NADP | 6.88×10^-3^ | 0.26 |
|  | 3 | *L*-aspartic acid | 4.14×10^-2^ | 0.26 |
|  | 4 | ciliatine | 4.53×10^-2^ | 0.27 |
|  | 5 | taurine | 2.72×10^-3^ | 0.27 |
|  | 6 | indoleacetic acid | 3.18×10^-2^ | 0.30 |
|  | 7 | guanidinosuccinic acid | 4.58×10^-2^ | 0.35 |
|  | 8 | 5-hydroxylysine | 1.62×10^-2^ | 0.38 |
|  | 9 | diaminopimelic acid | 2.58×10^-2^ | 0.43 |
|  | 10 | uridine 5'-diphospho-*N*-acetylgalactosamine | 2.67×10^-2^ | 0.43 |
|  | 11 | uridine diphosphate-*N*-acetylglucosamine | 2.67×10^-2^ | 0.43 |
|  | 12 | 3-methylcrotonyl-CoA | 3.95×10^-2^ | 0.52 |
|  | 13 | pyridoxamine | 4.91×10^-2^ | 0.56 |
|  | 14 | *L*-methionine | 4.87×10^-3^ | 1.80 |
|  | 15 | *N*-acetylneuraminic acid | 1.48×10^-2^ | 1.90 |
|  | 16 | *L*-palmitoylcarnitine | 1.15×10^-2^ | 2.12 |
|  | 17 | ethyl 3-indoleacetate | 3.37×10^-2^ | 2.15 |
|  | 18 | quinic acid | 6.76×10^-3^ | 2.20 |
|  | 19 | allantoin | 4.48×10^-2^ | 2.36 |
|  | 20 | guanosine diphosphate mannose | 9.00×10^-4^ | 3.33 |
|  | 21 | tetrahydrofolic acid | 3.43×10^-3^ | 4.22 |
|  | 22 | IMP | 3.29×10^-2^ | 7.18 |


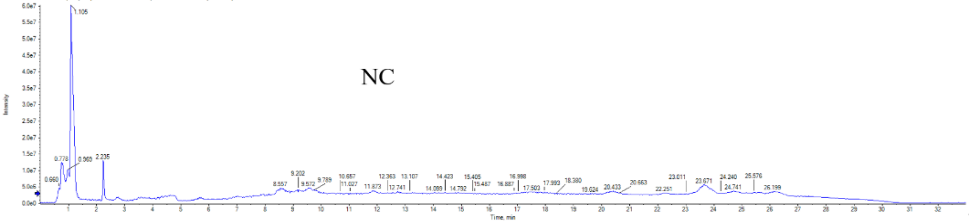

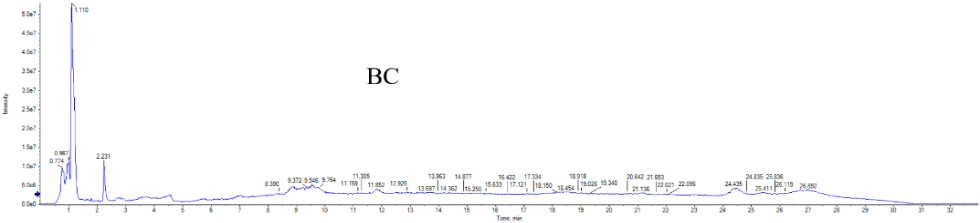

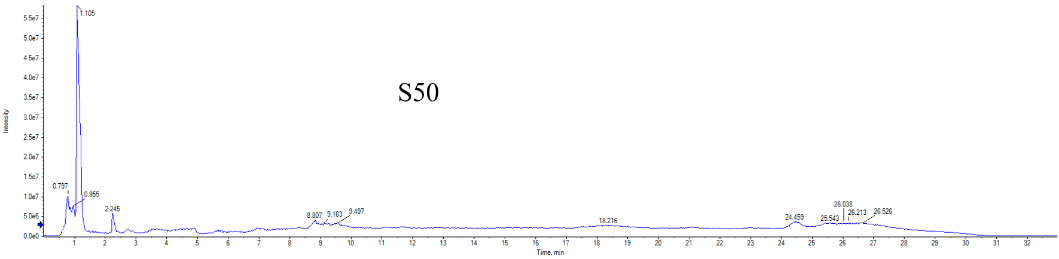

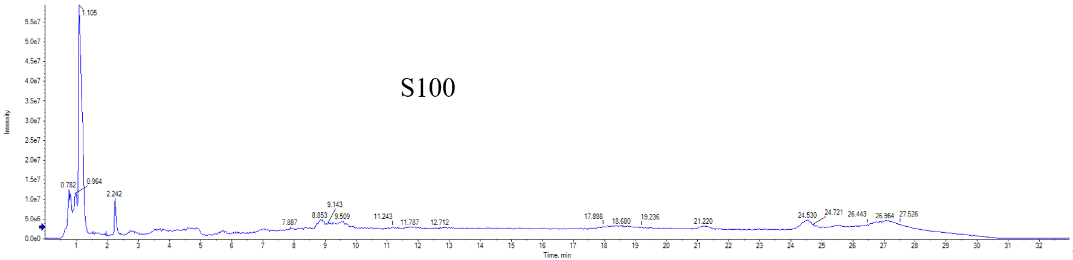

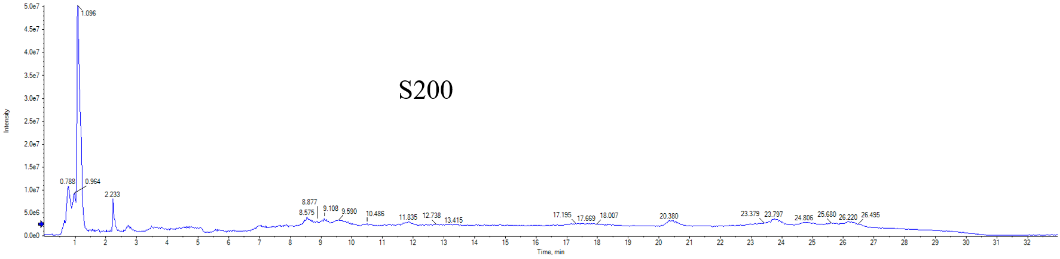


**(a)**


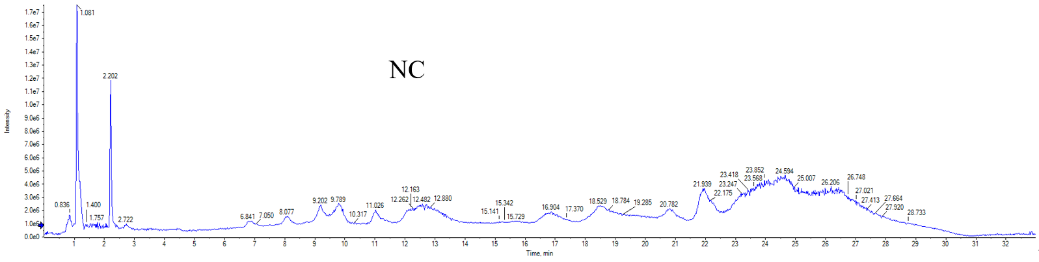

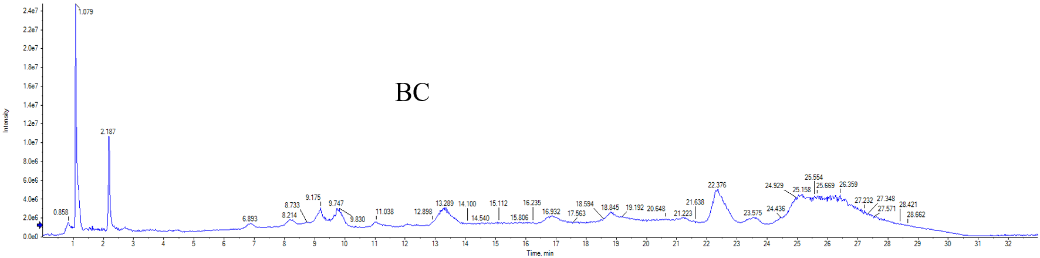

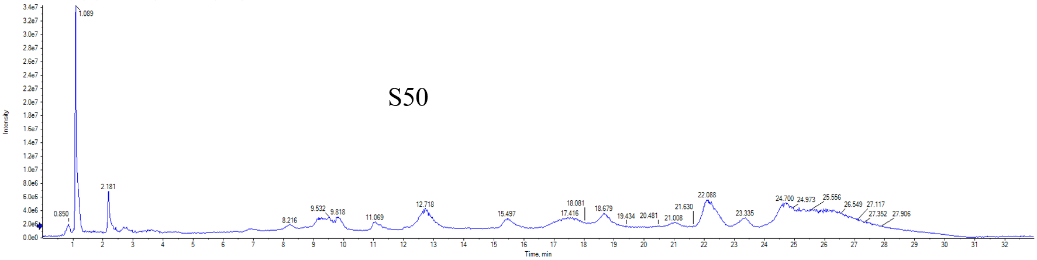

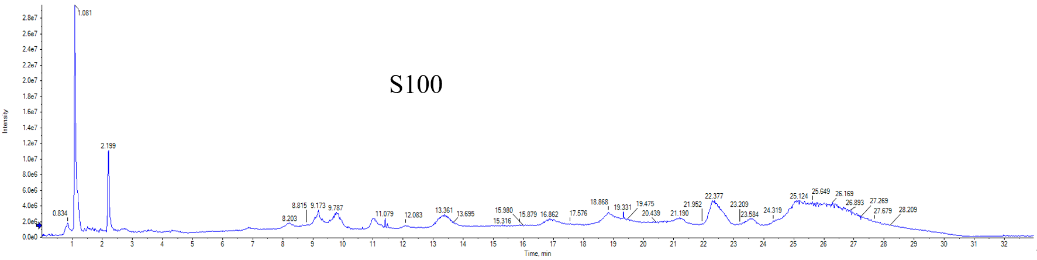

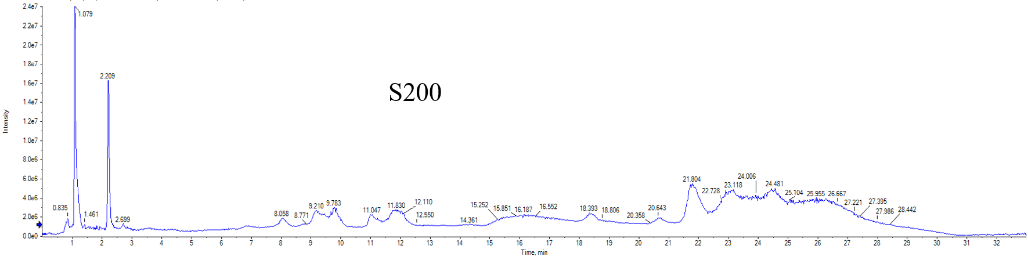


**(b)**

**Figure 1S** TICs of the feces samples (a) and the serum samples (b) from five groups (i.e., group BC, NC, S50, S100 and S200)

**S100 *vs* NC**


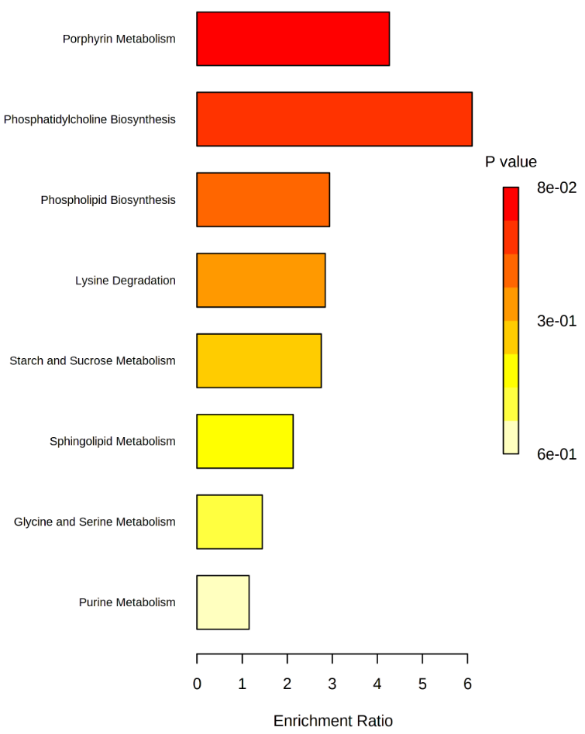

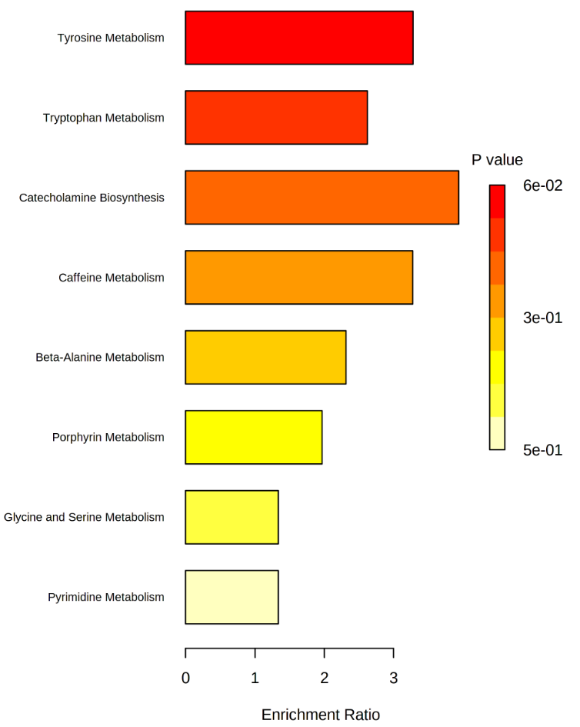


**S50 *vs* NC**

**S200 *vs* NC**


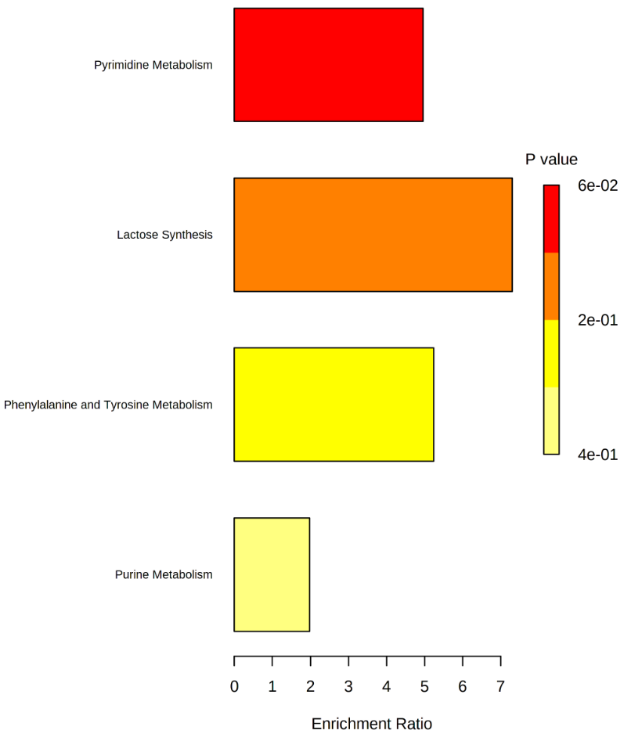


**Figure 2S** Overview of metabolites that were enriched on the groups of S50 *vs* NC, S100 *vs* NC and S200 *vs* NC based on feces intracellular metabolites of piglets, respectively.


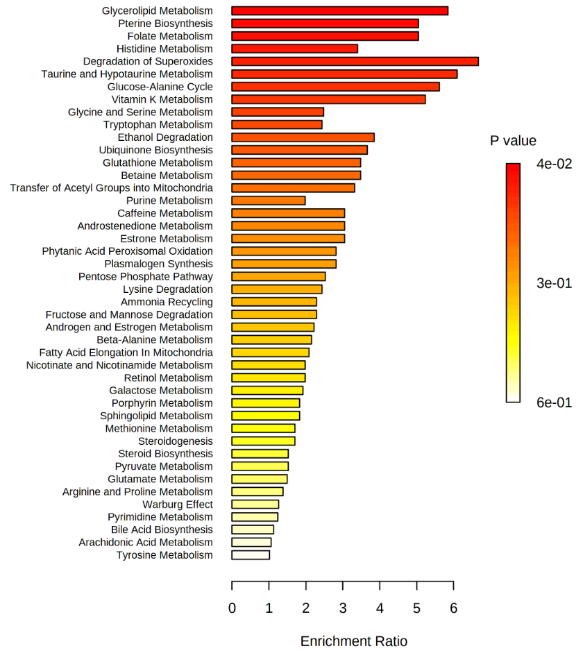


**S50 *vs* NC**


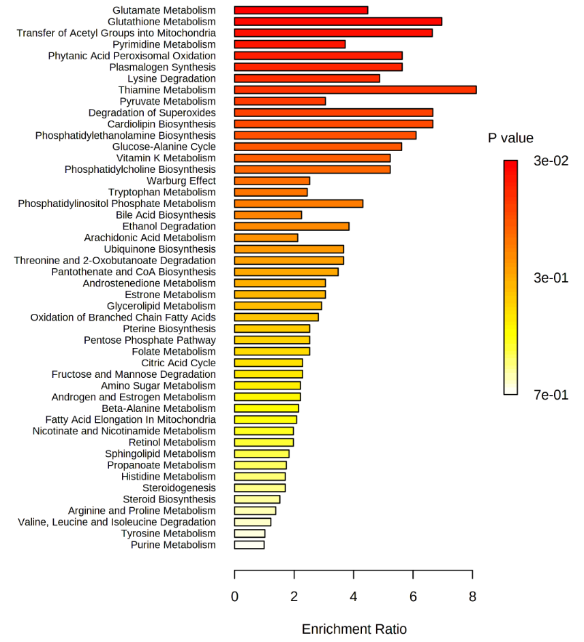


**NC *vs* BC**


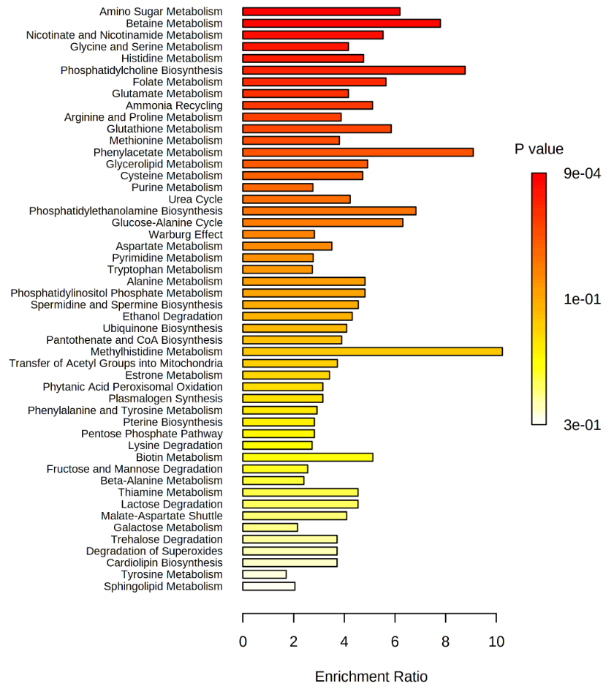


**S100 *vs* NC**


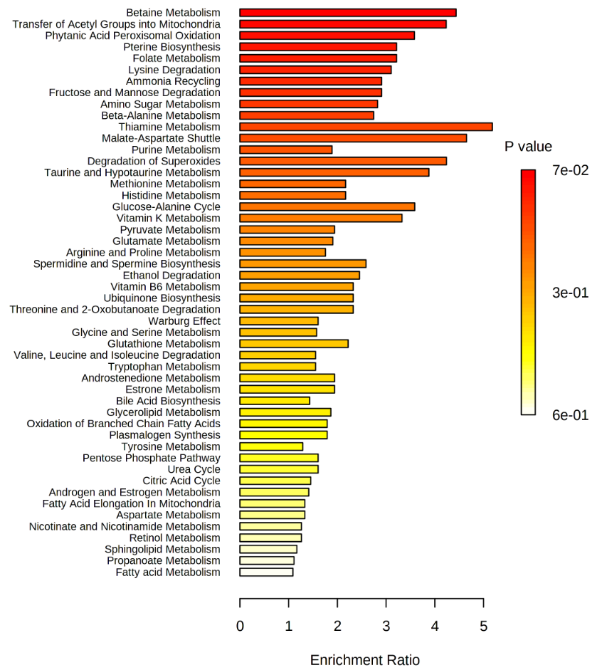


**S200 *vs* NC**

**Figure 3S** Overview of metabolites that were enriched on the groups of NC *vs* BC, S50 *vs* NC, S100 *vs* NC and S200 *vs* NC based on serum intracellular metabolites of piglets, respectively.
